# Complete Genome Sequence of Stenotrophomonas maltophilia Siphophage Silvanus

**DOI:** 10.1128/mra.01210-21

**Published:** 2022-02-28

**Authors:** Nancy Wang, James Garcia, Jay Clark, Tram Le, Ben Burrowes, Ry Young, Mei Liu

**Affiliations:** a Department of Biochemistry and Biophysics, Texas A&M University, College Station, Texas, USA; b Center for Phage Technology, Texas A&M University, College Station, Texas, USA; Portland State University

## Abstract

Stenotrophomonas maltophilia is an opportunistic Gram-negative bacterium capable of causing respiratory infections. S. maltophilia siphophage Silvanus was isolated, and its 45,678-bp genome is not closely related to known phages based on whole-genome comparative genomics analysis. It is predicted to use *cos*-type packaging due to the similarity of its large terminase subunit to that of phage HK97.

## ANNOUNCEMENT

Stenotrophomonas maltophilia is an emerging Gram-negative, multidrug-resistant pathogen most associated with respiratory infections in humans (1). With a goal of using phage as potential control for this pathogen, we report here the isolation and genome annotation of Silvanus, a siphophage targeting S. maltophilia.

Phage Silvanus was isolated from a soil sample collected from a horse pasture in College Station, TX (GPS coordinates 30°33′04.4″N, 96°18′44.0″W), in January 2019. Silvanus was isolated and propagated with the soft-agar overlay methods described previously (2) using an S. maltophilia strain (ATCC 51331) grown aerobically at 30°C in nutrient broth or agar (BD). Samples were negatively stained with 2% (wt/vol) uranyl acetate and imaged by transmission electron microscopy (TEM) at the Texas A&M Microscopy and Imaging Center (3). DNA was purified using a Promega Wizard DNA cleanup system as described (4), and the libraries were prepared using a Swift 2S Turbo library preparation kit and sequenced on an Illumina MiSeq machine with paired-end 150-bp reads and V2 300-cycle chemistry. The sequence reads were quality controlled with FastQC (www.bioinformatics.babraham.ac.uk/projects/fastqc) and trimmed with FASTX-Toolkit v0.11.6 (http://hannonlab.cshl.edu/fastx_toolkit/). Genomes were assembled from 85,453 trimmed reads with SPAdes v3.5.0 (5), and a contig with 138-fold sequencing coverage was obtained. The genome was closed by PCR and Sanger sequencing using forward primer 5′-CATCGTGTGTGGGCGAAATC-3′ and reverse primer 5′-TGAACCCCTGAGTTTCGTGG-3′. PhageTerm was used to predict phage termini from raw sequencing reads (6). The genome was assembled and annotated with the CPT Galaxy-Apollo phage annotation platform (https://cpt.tamu.edu/galaxy-pub) (7–9). Gene calling was conducted with GLIMMER v3 and MetaGeneAnnotator v1.0 (10, 11). tRNAs were detected with ARAGORN v2.36 and tRNAscan-SE v2.0 (12, 13). Gene function predictions were determined using InterProScan v5.48 (14) and BLAST v2.9.0 (15) against the NCBI nonredundant (nr) and Swiss-Prot databases (16), TMHMM v2.0 (17), HHPred, LipoP v1.0, and SignalP v5.0 (18–20). The genome-wide DNA sequence similarity to the top BLAST nucleotide hits was calculated with progressiveMauve v2.4 (21). All analyses were conducted at default settings.

**FIG 1:**
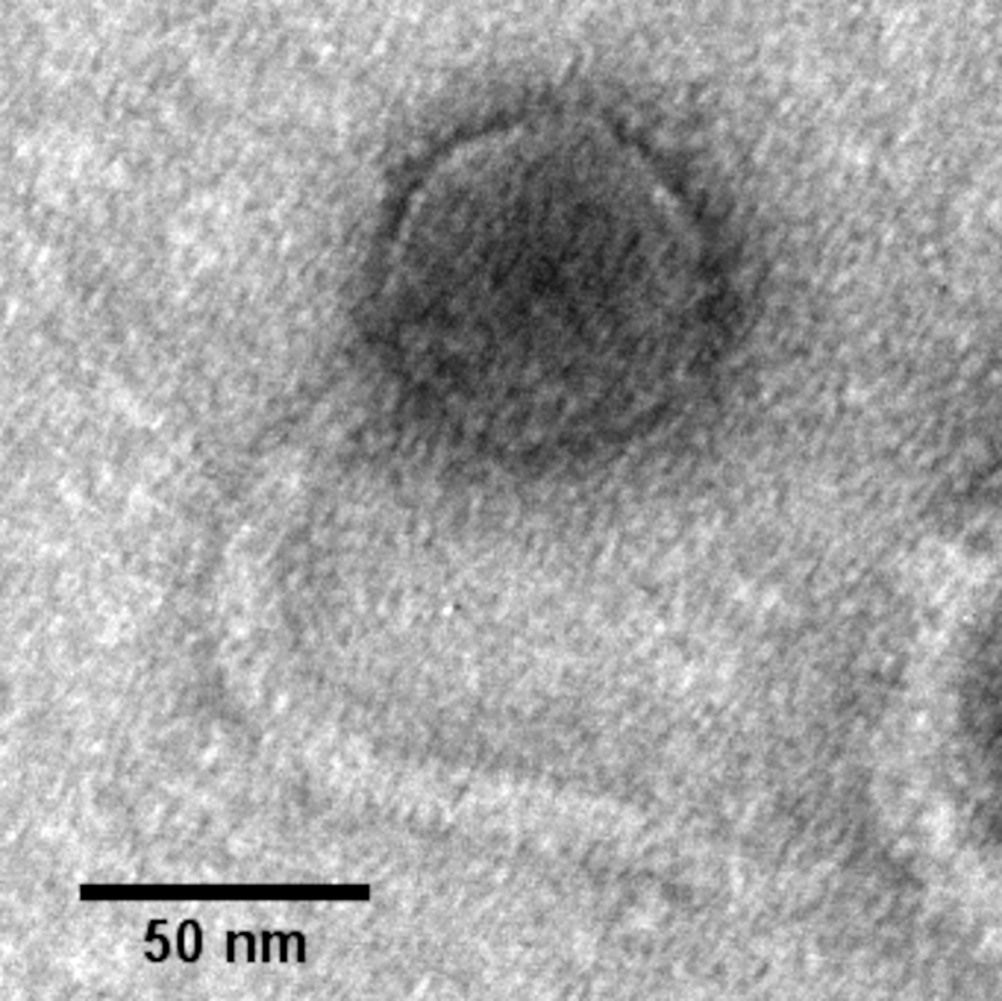
Transmission electron micrograph (TEM) of phage Silvanus. Phage particles were diluted with TEM buffer (20 mM NaCl, 10 mM Tris-HCl, pH 7.5, and 2 mM MgSO4) and captured on a freshly glow-discharged, Formvar carbon-coated grid. The grids were stained with 2% (wt/vol) uranyl acetate and observed on a JEOL 1200 EX TEM at 100 kV accelerating voltage at the Microscopy and Imaging Center at Texas A&M University.

Phage Silvanus has a siphophage morphology (Fig. 1). The 45,678-bp genome has a coding density of 97.4% and a G+C content of 58.4%. No tRNA genes were identified, and 26 out of 68 total genes were assigned putative functions, including a complete lysis cassette with genes encoding an endolysin of the glycosyl hydrolase class, a holin with three transmembrane domains and N-out, C-in topology (class I), and two-component spanins. Silvanus is predicted to use *cos*-type packaging because it encodes a large terminase subunit similar to that of the well-characterized *cos* phage HK97 (21% protein identity; E value, 10^−8^; 100% HHpred probability) and also encodes an HNH endonuclease similar to that of HK97 gp74 (40% protein identity; E value, 10^−21^) at the opposite end of the genome, which is required for the 3′ *cos* cleavage (22). Moreover, according to HHPred, the predicted small terminase has a 99.5% probability match to the structure of the Pseudomonas phage PaP3 small terminase, which generates cohesive ends (23). The precise location of phage Silvanus *cos* sites, however, cannot be determined by PhageTerm analysis. Whole-genome comparative genomics analysis by progressiveMauve v2.4 (21) revealed that Silvanus has <7% overall nucleotide identity to known phages. Silvanus was found to carry a T1 p38-like tail tape measure protein.

### Data availability.

The Silvanus genome was deposited in GenBank with accession number MZ326867. The associated BioProject, SRA, and BioSample accession numbers are PRJNA222858, SRR14095258, and SAMN18509682, respectively.
